# Editorial: Complexity and Connectivity: Functional Signatures of Neurodegenerative Disorders

**DOI:** 10.3389/fnins.2020.00916

**Published:** 2020-10-23

**Authors:** Carlos Gómez, Jesús Poza, Daniel Abásolo

**Affiliations:** ^1^Biomedical Engineering Group, University of Valladolid, Valladolid, Spain; ^2^Centro de Investigación Biomédica en Red en Bioingeniería, Biomateriales y Nanomedicina, (CIBER-BBN), Valladolid, Spain; ^3^IMUVA, Instituto de Investigación en Matemáticas, University of Valladolid, Valladolid, Spain; ^4^Department of Mechanical Engineering Sciences, Faculty of Engineering and Physical Sciences, Centre for Biomedical Engineering, University of Surrey, Guildford, United Kingdom

**Keywords:** connectivity, complexity, neurodegenerative diseases, signal and image processing, neuroimaging

In this Research Topic, we wanted to shed some light on the structure and function of one of the most complex biological systems: the human brain. Decades of research have helped to gain extensive knowledge about it. Advances in neuroimaging techniques, as well as intensive research on novel signal and imaging processing techniques, provide new findings that continuously update our knowledge on such an extraordinarily complex and dynamic system.

Neurodegenerative diseases affecting the central nervous system are a significant topic in brain research. In this regard, the papers included in this Research Topic analyzed diverse pathologies, such as dementia due to Alzheimer's disease (Cao et al.; Ferreira et al.; Huang et al.; Ponomareva et al.), amyotrophic lateral sclerosis (Wittstock et al.), frontotemporal dementia (Reyes et al.), Parkinson's disease (Prokic et al.), and cognitive impairment caused by white-matter hyperintensity (Yang et al.). Brain imaging techniques are of paramount importance in gaining knowledge on the complex underlying pathophysiology of these pathologies since they provide complementary information on brain structure, metabolism, and neural dynamics. This can be observed in the diverse techniques analyzed in this Research Topic: structural magnetic resonance imaging (sMRI), diffusion magnetic resonance imaging (dMRI), positron emission tomography (PET), functional magnetic resonance imaging (fMRI), transcranial magnetic stimulation (TMS), magnetoencephalography (MEG), and electroencephalography (EEG). These contributions reinforce the need for multimodal approaches to further understand the multifaceted nature of the neurodegenerative processes involved in these pathologies.

An example of this multimodal approach is presented in the study by Ponomareva et al., in which the combination of genetics, MRI, and EEG helped to further understand the complexity of neurodegeneration associated with dementia due to Alzheimer's disease (AD). Specifically, they explored the influence of the *PICALM* rs3851179 genotype on brain structure and function in non-demented subjects. Their results suggest that the protective *PICALM* rs3851179 allele prevents cognitive decline by promoting a positive association between alpha brain connectivity and the corpus callosum size (Ponomareva et al.). The importance of the relationship between structural and functional brain networks in dementia due to AD is also evidenced in the study by Cao et al.. This research found that AD and mild cognitive impairment (MCI) exhibit an abnormal increase in the structural-functional coupling, in terms of the rich-club structure of the brain network. Previous studies also support the intricate pathophysiology associated with AD, which involves a complex and multifaceted pattern of alterations in genetic, structural, functional, and also, metabolic networks.

Metabolic brain networks play an important role in obtaining a wide perspective of neurodegenerative processes in AD. In an interesting study, Huang et al. presented a novel methodology to build individual metabolic brain networks based on 18F-FDG PET imaging, rather than using a conventional group-based approach. The proposed method was successfully applied to characterize the different stages of dementia due to AD, supporting the idea that neurodegenerative processes associated with AD induce progressive changes in the metabolic network. Furthermore, Huang et al. found that the results obtained with the individual metabolic brain networks are aligned with those obtained using the groupwise network methodology. Group-level analyses are certainly very useful in obtaining representative patterns of AD stages; however, they pose an interesting question: does AD staging accurately represent the multifaceted, heterogeneous nature of the disease? Dementia due to AD is certainly a heterogeneous disease. In Ferreira et al., different subtypes of AD were related to distinct patterns of brain atrophy. These abnormalities were quantified by computing the structural MRI network and analyzing diverse network properties (i.e., integration, segregation, and modularity). The particular structural network signature of each AD subtype poses a new challenge in the redefinition of this neuropathology and raises important clinical implications that should be further investigated.

Although AD has received much attention in the last decades due to its very high prevalence, understanding of other neurodegenerative diseases is also essential to ensuring early diagnosis, as well as an appropriate and timely treatment. There is also a high incidence of Parkinson's disease (PD) mainly among the elderly population, and it is anticipated that there will be a dramatic increase in the number of people suffering from it due to increased life expectancy in Western countries. With the aim of providing a better understanding of PD, Prokic et al. studied the effects of zolpidem on MEG activity during the performance of a motor finger-tapping task. The study shows that during the task, there is a cumulative increase of beta oscillations in the primary motor cortex, which is reduced following zolpidem, but not placebo. Zolpidem also produced a significant decrease in reaction time and an increase in tapping speed. Authors concluded that GABAergic modulation in PD bradykinesia plays an important role, which merits further exploration as a therapeutic target (Prokic et al.).

Much less common than AD and PD are the remaining three neurodegenerative disorders analyzed in this Research Topic: amyotrophic lateral sclerosis (ALS), cognitive impairment associated with white matter hyperintensity (WMH), and fronto-temporal dementia (FTD). These three studies (Reyes et al.; Wittstock et al.; Yang et al.) share a common factor: in all of them, diffusion tensor imaging (DTI) data were analyzed. The functional and structural alterations of callosal integrity in ALS patients was assessed using DTI by Wittstock et al.. Results show that TMS produced a longer latency of the ipsilateral silent period in ALS patients than in controls. Additionally, the fractional anisotropy values of the corticospinal tract were significantly lower in ALS patients compared with controls (Wittstock et al.). In another study, Yang et al. carried out a graph-based analysis to explore the relationships between WMH and cognitive impairment. To explore this, Yang et al. analyzed the properties of the structural brain network, constructed using DTI fiber tractography. Graph-based parameters showed that WMH causes alterations in diverse network features (i.e., efficiency, segregation, and global connectivity), with these being more important in subjects with cognitive impairment. Finally, DTI was used to identify atrophy patterns of white matter in different FTD variants (Reyes et al.). DTI images revealed damage in the internal networks of the left temporal lobe for the group of patients with the semantic variant of primary progressive aphasia. On the other hand, atrophy in networks from the basal ganglia to the motor and premotor areas was reported for the non-fluent/agrammatic group. These findings can help gain a better understanding of the cognitive model of speech/language and their alterations in neurodegenerative diseases.

The contributions to this Research Topic fill some of the gaps existing between complexity and connectivity analyses for the characterization of complex dynamics in brain networks and the identification of the functional significance of different neurodegenerative disorders. In addition to being worthy contributions to the state of the art, they could be the starting point of novel developments in the field that would contribute to mitigating the challenges posed by these disorders as a result of increased life expectancy. We hope that the reader will find the publications included in this Research Topic useful and stimulating.

## Author Contributions

CG, JP, and DA wrote the manuscript. All authors contributed to the article and approved the submitted version.

## Conflict of Interest

The authors declare that the research was conducted in the absence of any commercial or financial relationships that could be construed as a potential conflict of interest.

